# Food Matrix Effects of Polyphenol Bioaccessibility from Almond Skin during Simulated Human Digestion

**DOI:** 10.3390/nu8090568

**Published:** 2016-09-15

**Authors:** Giuseppina Mandalari, Maria Vardakou, Richard Faulks, Carlo Bisignano, Maria Martorana, Antonella Smeriglio, Domenico Trombetta

**Affiliations:** 1 Dipartimento di Scienze Chimiche, Biologiche, Farmaceutiche ed Ambientali, University of Messina, Sal. Sperone 31, 98166 Messina, Italy; cbisignano@unime.it (C.B.); mmartorana@live.it (M.M.); asmeriglio@unime.it (A.S.); dtrombetta@unime.it (D.T.); 2 The Model Gut, Institute of Food Research, Norwich Research Park, Colney Lane, Norwich NR4 7UA, UK; M.Vardakou@uea.ac.uk (M.V.); r.faulks147@btinternet.com (R.F.)

**Keywords:** almond skin, food matrix, simulated human digestion, polyphenols, bioaccessibility

## Abstract

The goal of the present study was to quantify the rate and extent of polyphenols released in the gastrointestinal tract (GIT) from natural (NS) and blanched (BS) almond skins. A dynamic gastric model of digestion which provides a realistic simulation of the human stomach was used. In order to establish the effect of a food matrix on polyphenols bioaccessibility, NS and BS were either digested in water (WT) or incorporated into home-made biscuits (HB), crisp-bread (CB) and full-fat milk (FM). Phenolic acids were the most bioaccessible class (68.5% release from NS and 64.7% from BS). WT increased the release of flavan-3-ols (*p* < 0.05) and flavonols (*p* < 0.05) from NS after gastric plus duodenal digestion, whereas CB and HB were better vehicles for BS. FM lowered the % recovery of polyphenols, the free total phenols and the antioxidant status in the digestion medium, indicating that phenolic compounds could bind protein present in the food matrix. The release of bioactives from almond skins could explain the beneficial effects associated with almond consumption.

## 1. Introduction

The presence of polyphenols in almond skin has been related to several health benefits associated with almond (Prunus dulcis Miller D.A. Webb) consumption [1,2,3]. The antioxidant and free-radical scavenging activity of almond skin polyphenols has been reported [4]. It has been shown that flavonoids and phenolic acids, including flavonols, flavanols, flavanones and simple phenolic acids identified in almond skins may play a role in reducing risk factors against chronic inflammatory diseases and ageing disorders [5,6]. A range of biological effects of flavonoids, including anticancer, antiviral, antimutagenic and anti-inflammatory activities, have been reported [7,8]. Nevertheless, one of the major limiting factors affecting the beneficial effects of polyphenols is their bioaccessibility and subsequent absorption in the gastrointestinal tract (GIT), together with their bio-transformation by the gut microbiota enzymes [9].

This process depends on the physico-chemical properties of the food matrix and its changes during digestion. We refer to bioaccessibility as the proportion of a nutrient or phytochemical compound ‘released’ from a complex food matrix during digestion and therefore becoming potentially available for absorption in the GIT. A number of studies have reported that food matrix affects polyphenol release in the gut as well as the efficacy by which they are transported across the mucosal epithelium [10,11]. The presence of a food matrix (muffin) decreased the bioaccessibility of certain bioactive compounds, such as protocatechuic acid and luteolin, from raw shelled and roasted salted pistachios during simulated human digestion [12]. Interaction with other food nutrients and the formation of complexes mainly with protein and fat is also known to affect bioaccessibility of phenolic acids [13]. The influence of digestion conditions, such as pH, temperature, bile salts, gastric and pancreatic enzymes on the bioaccessibility of certain polyphenols has been reported [14,15]. Milk has been found to affect bioaccessibility of epicatechin metabolites [16]. We have previously identified a combination of flavonols, flavan-3-ols, hydroxybenzoic acids and flavanones present in almond skin [1]: the major flavonoids were (+)-catechin, (−)-epicatechin, kaempferol and isorhamnetin, both as aglycones or conjugated with rhamnose (Rha) and glucose (Glc). The total phenolic content, expressed as mg gallic acid equivalents (GAE) per 100 g of fresh skin, was higher in natural almond skin (NS, 3474.1 ± 239.8) than blanched almond skin (BS, 278.9 ± 12.0). The blanching process is known to remove most of the water-soluble flavonoids and other polyphenols [1]. BS, obtained by industrial blanching, currently represents a commercially available product. Our previous investigation on the release of almond skin polyphenols during simulated human digestion using a static model demonstrated higher percentages of polyphenols released from NS compared to BS [17].

The aim of the present study was to assess the effect of a range of food matrices on the rate and extent of polyphenol bioaccessibility from NS and BS during simulated human digestion. A dynamic gastric model (DGM) was used to simulate the human stomach [12,18]. Gastric digesta were then subjected to a duodenal phase in order to simulate the full human upper GIT.

## 2. Materials and Methods 

### 2.1. Production of Test Meals

Natural almonds with intact skin were kindly provided by the Almond Board of California and stored in the dark. NS was removed using liquid-nitrogen as previously reported and milled [17]. BS, provided by ABCO laboratories, was obtained by hot water blanching, dried and powdered. Home-made biscuits (HB) containing NS or BS were prepared using the following ingredients: white flour (200 g), butter at room temperature (100 g), sugar (sucrose, 100 g), eggs (one standard egg) and baked at 180 °C for 12 min. For the digestion experiments, 25 g of HB containing 2 g of either NS or BS were used. Home-made crisp-bread (CB) containing NS or BS was prepared using the following ingredients: baking soda (5 g), hot water (400 mL), salt (1.2 g), fennel seed (1 g), white flour (250 g) and baked at 230 °C for 2–4 min. For the digestion experiments, 34 g of CB containing 2 g of either NS or BS were used.

### 2.2. Chemicals and Enzymes

Egg *L*-α-phosphatidylcholine (PC, lecithin grade 1, 99% purity) was obtained from Lipid Products (South Nutfield, Surrey, UK). Porcine gastric mucosa pepsin, bovine α-chymotrypsin, pancreatic α-amylase, porcine colipase, porcine pancreatic lipase and bile salts were obtained from Sigma (Poole, Dorset, UK). Lipase for the gastric phase of digestion was a gastric lipase analogue of fungal origin (F-AP15) from Amano Enzyme Inc. (Nagoya, Japan). All flavonoid and other phytochemical standards were obtained from either Sigma-Aldrich (Poole, UK) or Extrasynthese (Genay, France). All solvents were HPLC grade, water was ultra-pure grade, and other chemicals were of AR quality.

### 2.3. Simulated Human Digestion

Eight meals were prepared as follows and subjected to in vitro gastric and gastric plus duodenal digestion: WT (200 mL) containing either NS (2 g) or BS (2 g), HB (25 g) containing either NS (2 g) or BS (2 g) added to water (240 mL), CB (34 g) containing either NS (2 g) or BS (2 g) added to water (240 mL), FM (200 mL) containing either NS (2 g) or BS (2 g). 

### 2.4. Gastric Digestion

Individual meals were fed onto the DGM in the presence of priming acid (20 mL), as previously reported [18]. In order to replicate the conditions found in the human stomach, samples were processed in two zones: within the fundus/main body of the DGM, where the meals were subjected to inhomogeneous mixing while gastric acid and enzyme secretions were added; in the antrum, where physiological shear and grinding forces were applied in order to mimic the antral shearing and rate of delivery to the duodenum. The composition of the simulated gastric acid solution has also been previously reported [12]. The simulated gastric enzyme solution was prepared by dissolving porcine gastric mucosa pepsin and a gastric lipase analogue from *Rhizopus oryzae* in the above described salt mixture (no acid) at a final concentration of 9000 U/mL and 60 U/mL for pepsin and lipase, respectively. A suspension of single-shelled lecithin liposomes was added to the gastric enzyme solution at a final concentration of 0.127 mM. 

A total of six samples (G1–G6) were ejected from the antrum of the DGM at regular intervals during each run (see Table 1 for sampling details) in order to replicate the predicted gastric emptying regimes under physiological conditions. Samples digested in WT were ejected from the antrum of the DGM every 4 min: the amount of gastric acid secretion was 1.5 ± 0.1 mL and 1.6 ± 0.1 mL for NS and BS respectively; the amount of gastric enzyme secretion was 2.8 ± 0.1 mL and 2.7 ± 0.1 mL for NS and BS respectively. Samples digested in HB were ejected from the antrum of the DGM every 4 min: the amount of gastric acid secretion was 6.4 ± 0.1 mL and 6.3 ± 0.1 mL for NS and BS respectively; the amount of gastric enzyme secretion was 11.2 ± 0.2 mL and 11.4 ± 0.1 mL for NS and BS respectively. Samples digested in CB were ejected from the antrum of the DGM every 5 min: the amount of gastric acid secretion was 17.6 ± 0.2 mL and 18.2 ± 0.2 mL for NS and BS respectively; the amount of gastric enzyme secretion was 13.8 ± 0.1 mL and 14.2 ± 0.2 mL for NS and BS respectively. Samples digested in FM were ejected from the antrum of the DGM every 6 min: the amount of gastric acid secretion was 4.4 ± 0.2 mL and 4.6 ± 0.2 mL for NS and BS respectively; the amount of gastric enzyme secretion was 13.1 ± 0.3 mL and 13.8 ± 0.2 mL for NS and BS respectively. A control digestion without addition of gastric enzymes was performed for each meal. Each gastric sample was weighed, its pH recorded and adjusted to 7.0 with NaOH (1 M) in order to inhibit gastric enzyme activity. 

### 2.5. Duodenal Digestion

Individual gastric samples (23 g, G1 to G6) were transferred upon ejection, to a Sterilin plastic tube for duodenal digestion with the addition of simulated bile solution (2.5 mL) and pancreatic enzyme solution (7.0 mL) and incubated at 37 °C under shaking conditions (170 rpm) for 2 h. Simulated bile was prepared fresh daily. It contained lecithin (6.5 mM), cholesterol (4 mM), sodium taurocholate (12.5 mM), and sodium glycodeoxycholate (12.5 mM) in a solution containing NaCl (146.0 mM), CaCl2 (2.6 mM) and KCl (4.8 mM). 

Pancreatic enzyme solution contained NaCl (125.0 mM), CaCl_2_ (0.6 mM), MgCl_2_ (0.3 mM), and ZnSO_4_·7H_2_O (4.1 μM). Porcine pancreatic lipase (590 U/mL), porcine colipase (3.2 μg/mL), porcine trypsin (11 U/mL), bovine α-chymotrypsin (24 U/mL) and porcine α-amylase (300 U/mL) were added to the pancreatic solution. 

### 2.6. Poliphenols Extraction from Samples before and after Dynamic in Vitro Digestion

All original samples (WT, HB, CB and FM containing NS or BS) and aliquotes obtained from each sample subjected to a dynamic in vitro gastric digestion (NSWT G, NSHB G, NSCB G, NSFM G, BSWT G, BSHB G, BSCB G, BSFM G) and gastric plus duodenal digestion (NSWT G + D, NSHB G + D, NSCB G + D, NSFM G + D, BSWT G + D, BSHB G + D, BSCB G + D, BSFM G + D), were harvested and centrifuged to separate the residual material from the supernatant. The volume of each supernatant was measured; the residues were dried in a forced air heated oven (T °C < 40 °C) and brought to constant weight.

Each residue was extracted with hexane (1:5, w/v) to remove the lipid fraction. The procedure was repeated 3 times. Afterwards it was extracted with a methanol/water mixture (70:30) (1:10, w/v) by shaking for 5 min and sonicating for 10 min. After centrifugation at 12,074 rcf for 10 min, the supernatant was collected. The procedure was repeated 3 times. The supernatants were pooled. In order to precipitate proteins, MeOH (8 mL) and 2M NaOH (600 µL) were added in 10 mL extract. Samples were stirred vigorously and after centrifugation at 5916 rcf for 5 min the supernatant was brought to dryness in a rotavapor. Finally, the residue was resuspended with 10 mL of 1% HCl in MeOH and extracted, using a separatory funnel, with the same volume of ethyl acetate. The extraction was repeated 4 times. The ethyl acetate fractions were combined and evaporated to dryness in a rotavapor. The residue was weighed, solubilised in MeOH, filtered through a Nalgene 0.22 μM nylon filter and subjected to total phenol, radical scavenging activity and HPLC analysis.

For NS and BS digested in water no protein precipitation step was perfermed, given that they were not incorporated into any food matrix.

### 2.7. Polyphenols Release and Radical Scavenging Activity

Total phenol content was determined colorimetrically by the Folin-Ciocalteu method as modified by Singleton, Orthofer and Lamuela-Raventos [19] using gallic acid as a reference compound. Total phenol content was expressed as mg of gallic acid equivalents (GAE) per 100 g of sample. The anti-radical activity was determined using the stable 2,2-diphenyl-1-picrylhydrazyl radical (DPPH) and the procedure previously described [20]. Results were expressed as mg of extract needed to scavenge 50 µmol of the initial DPPH concentration (SE50). The determination of phenolics and flavonoids was carried out using a Shimadzu high performance liquid chromatography system equipped with an UV–Vis photodiode-array detector (DAD) (SPD-M10AVP, Shimadzu, Kyoto, Japan) and a fluorescence detector (1046A Hewlett Packard, Palo Alto, CA, USA), as previously reported [17].

### 2.8. Statistical Analysis

All assays were performed in triplicate and expressed as means ± standard deviation (SD). Data analysis was performed using ANOVA tests using SigmaPlot software version 12.0 for Windows (SPSS Inc., Hong Kong, China). To isolate the group or groups that differ from the others, a multiple comparison procedure (*Tukey* Test) was used. Results were considered statistically significant at *p* < 0.05.

## 3. Results

### 3.1. Polyphenols Release during Simulated Digestion

The polyphenolic content of the baseline meals (NS WT, NS HB, NS CB and NS FM, BS WT, BS HB, BS CB and BS FM) is reported in Table 2. As expected, the NS meals had a total phenol content nearly ten times higher than the BS meals. 

**Figure 1:**
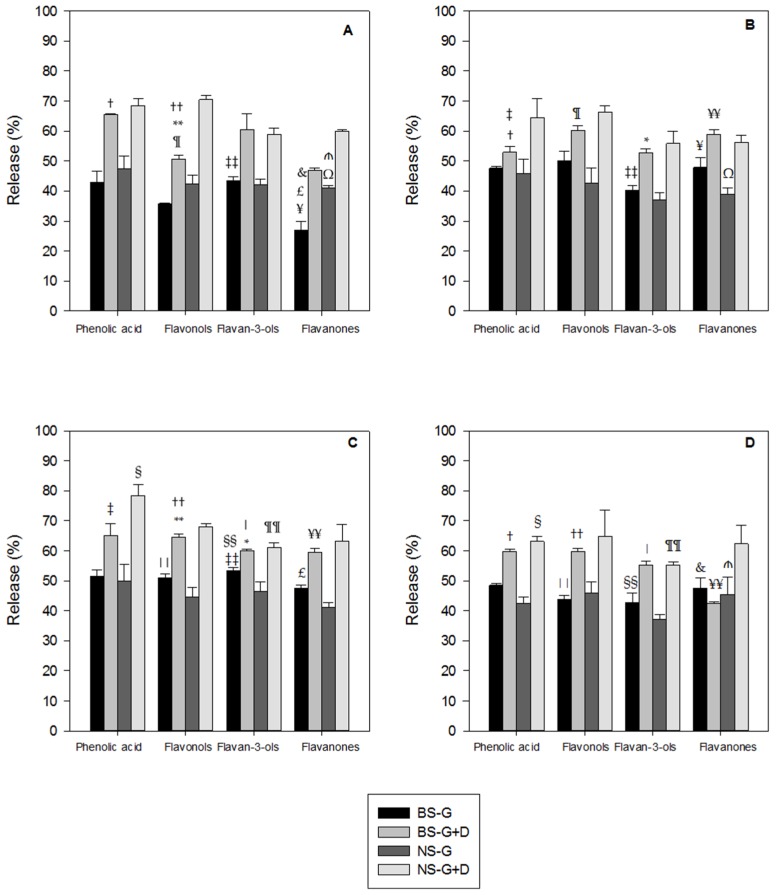
Release of flavonoids and phenolic acids from natural almond skin (NS) and blanched almond skin (BS) in water (**A**); home-made biscuits (**B**); crisp-bread (**C**) and full-fat milk (**D**). Values are given as % phenolic acids, flavanols, flavan-3-ols and flavanones released from the initial amounts presents in the meals (Table 2) during in vitro gastric (G) and gastric + duodenal (G + D) digestion. Values represent averages (±SD) of triplicate measurements. Matching symbols across the four panels indicate significantly different (*p* < 0.01) samples. ^†^ Phenolic acid release in BS-G + D significantly different between A, B and D; ^††^ Flavonols release in BS-G + D significantly different between A, C and D; ** Flavonols release in BS-G + D significantly different between A and C; ^¶^ Flavonols release in BS-G + D significantly different between A and B; ^‡‡^ Flavan-3-ols release in BS-G significantly different between A, B and C; ^&^ Flavanones release in BS-G significantly different between A and D; ^¥^ Flavanones release in BS-G significantly different between A and B; ^ѫ^: Flavanones release in NS-G significantly different between A and D; ^Ω^ Flavanones release in NS-G significantly different between A and B; ^‡^ Phenolic acid release in BS-G + D significantly different between B and C; * Flavan-3-ols release in BS-G + D significantly different between B and C; ^¥¥^ Flavanones release in BS-G + D significantly different between B, C and D; ^§^ Phenolic acid release in NS-G + D significantly different between C and D; ^||^ Flavonols release in BS-G significantly different between C and D; ^§§^ Flavan-3-ols release in BS-G significantly different between C and D; ^|^ Flavan-3-ols release in BS-G + D significantly different between C and D; ^¶¶^ Flavan-3-ols release in NS-G+D significantly different between C and D.

The release of polyphenols as a percentage of the original amount present in each meal (Table 2) after simulated gastric plus duodenal digestion is reported in Figure 1. No polyphenols were detected in blank samples of each meal not containing almond skin. As expected, a high release of bioactive compounds was observed from both NS and BS in WT (Figure 1A).

**Figure 2:**
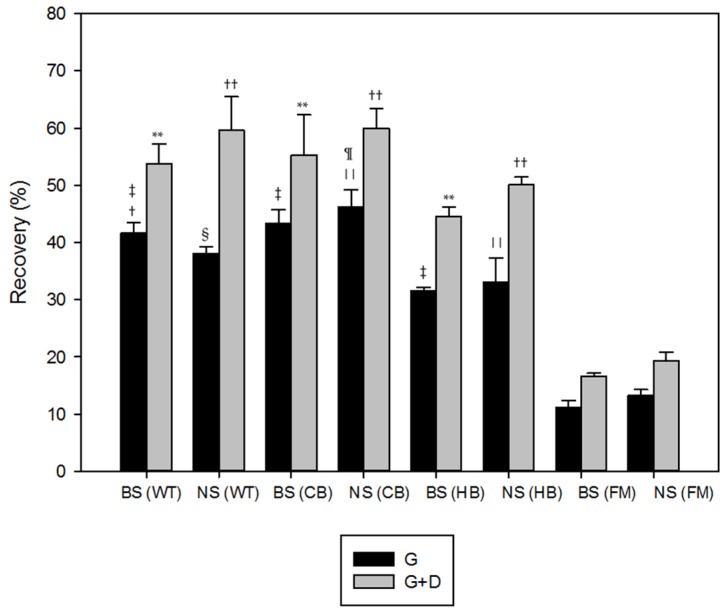
Recovery of total phenolic compounds in the digestion medium from natural almond skin (NS) and blanched almond skin (BS) in water (WT), home-made biscuits (HB), crisp-bread (CB) and full-fat milk (FM). Values are given as % of total phenolic compounds calculated from the amount of polyphenols present in the medium at the end of each step of digestion. Values represent averages (±SD) of triplicate measurements. ^†^ *p* < 0.01 vs. BS (CB); ^‡^ *p* < 0.01 vs. BS (FM); ^§^ *p* < 0.01 vs. NS (CB); ^||^ *p* < 0.01 vs. NS (FM); ^¶^ *p* < 0.01 vs. NS (HB); ** *p* < 0.01 vs. BS (FM); ^††^ *p* < 0.01 vs. NS (FM).

The % release from NS and BS in WT during the gastric phase of digestion was higher for phenolic acids (47.1% from NS and 45.3% from BS) compared with the other classes of polyphenols, with a further increase in the duodenal phase of digestion (68.5% from NS and 64.7% from BS). Lower % release from BS in WT was observed with flavanones after both gastric (29.3%) and gastric plus duodenal incubation (48.2%). Higher release of flavonols (65.6%) and phenolic acids (59.4%) was observed after in vitro gastric plus duodenal digestion from NS (Figure 1A). The % of recovery, calculated from the amount of polyphenols present in the medium at the end of each step of digestion, confirmed the data obtained from the % of release (Figure 2). This data demonstrated a different bioaccessibility across the various classes of polyphenols in the absence of an interfering food matrix. In accordance with our previous investigation [1], high release of polyphenols was detected when NS and BS were incubated in WT. However, the static and dynamic digestion models used affected the rate and extent of bioactives potentially available for absorption in the gut.

The % of release and recovery of polyphenols from NS and BS incorporated into HB are reported in Figure 1B and Figure 2, respectively. Phenolic acids were the class of polyphenols mostly released from NS in the gastric phase, followed by flavonols and flavanones, with an average % release of 40.72 in the gastric compartment. Flavonols had the highest % release from BS in the gastric phase (48.5), followed by flavanones and phenolic acids. For both NS and BS, the gastric + duodenal digestion (G + D) produced only a slight increase in polyphenol release over that observed in the gastric compartment. In addition, higher percentages of phenolic acids and flavonols were released from NSHB G + D. 

Higher % of release of phenolic acids, flavonols, flavan-3-ols and flavanones were observed in BSCB G compared with NSCB G, whereas the opposite behaviour was detected in the duodenal phase (Figure 1C). A higher release of phenolic acids was observed in BSCB G (52.7%) compared with BSWT G (45.3%), as well as flavonols both in the gastric (51.4 in BSCB G vs. 33.9 in BSWT G) and in the duodenal phase (63.9 in BSCB G + D vs. 52.3 in BSWT G + D) and flavanones both in the gastric (47.6 in BSCB G vs. 29.3 in BSWT G) and in the duodenal phase (59.8 in BSCB G + D vs. 48.2 in BSWT G + D). The % release data were confirmed by % recovery values (Figure 2). 

The % of release and recovery of flavonoids and phenolic acids from NS and BS incorporated into FM are reported in Figure 1D and Figure 2, respectively. The highest % release from NS was detected with flavonols after gastric plus duodenal digestion, followed by flavanones and phenolic acids. About 60% of phenolic acids and flavanones were released from BS in FM after simulated digestion.

Statistical analysis of Figure 1 showed significant differences in the % of bioactives released from the tested meals: higher release (*p* < 0.05) of phenolic acid was detected from NS in CB vs. FM after G + D, flavan-3-ols from NS in WT vs. FM after G + D and flavonols from NS in WT vs. HB after G + D; higher release (*p* < 0.01) of flavan-3-ols was also observed from NS in CB vs. FM after G + D; higher release (*p* < 0.05) of flavan-3-ols was found from NS in CB vs. FM after G; higher release (*p* < 0.01) of flavanones from BS in HB vs. WT after G and vs. FM after G + D and flavan-3-ols from BS in CB vs. HB after G + D; higher release (*p* < 0.01) of flavanones from BS in FM, CB and HB vs. WT after G + D and of flavonols in CB vs. FM after G; higher release (*p* < 0.05) of phenolic acids from BS in CB vs. WT after G. This data confirmed the presence of a food matrix affected the release of bioactives from almond skin during simulated digestion. Overall WT and CB were good vehicles for the release of polyphenols from NS, whereas HB and CB were optimal for polyphenols bioaccessibility from BS. This could be due to the previous loss of water soluble polyphenols from BS during the industrial blanching process.

Statistical analysis of Figure 2 showed significant differences in the % recovery of polyphenols from the tested meals: higher recovery was observed from both NS and BS in CB vs. HB (*p* < 0.05) and vs. FM (*p* < 0.01) after G + D; higher recovery was also detected from NS in CB vs. WT (*p* < 0.01), FM (*p* < 0.01) and HB (*p* < 0.01) after G, as well as NS in WT vs. HB (*p* < 0.05); lower recovery (*p* < 0.01) was observed with BS in WT vs. CB and FM vs. HB after G. The recovery data also confirmed WT was a good vehicle for bioactives bioaccessibility from NS, whereas CB was optimal for BS.

Significantly (*p* < 0.01) lower % recoveries were obtained with FM from both NS and BS after gastric plus duodenal digestion. We believe the % recovery demonstrated that milk protein were able to bind the free polyphenols present in the digestion medium and the interaction between phenolic compounds and proteins was more pronounced in milk rather than biscuits [21]. Furthermore, complexes made by protein and tannin are less well digested and the amino acid profile may be damaged. 

**Figure 3:**
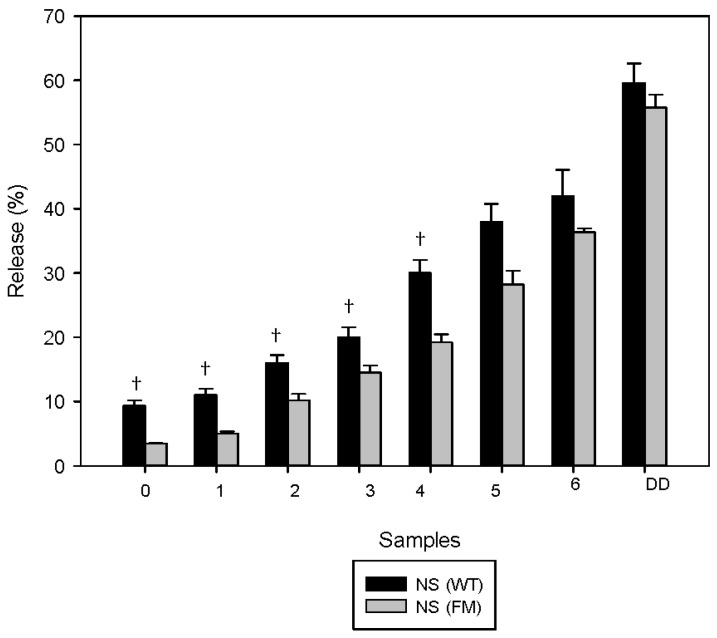
Kinetic of release of flavan-3-ols from natural almond skin (NS) in water and full-fat milk. Values are given as % of flavan-3-ols released from the initial amounts of flavan-3-ols in the meals (Table 2) during in vitro gastric (samples 0 to 6, see Table 1 for sampling time) and gastric + duodenal (DD) digestion. Values represent averages (±SD) of triplicate measurements. SD were always <10%. Water (WT), full-fat milk (FM). ^†^ *p* < 0.01 vs. NS (FM).

The same trend was also detected when comparing the kinetics (%) of release for flavan-3-ols from NS in WT and FM during the full digestion process (Figure 3). When NS was digested in WT, a steady release of flavan-3-ols over time was observed, corresponding to a significant increase detected for the initial 5 gastric digestion samples. In the presence of FM, the dynamics of release were very similar to WT but at a significantly slower rate.

### 3.2. Antioxidant Profile during Digestion

**Figure 4:**
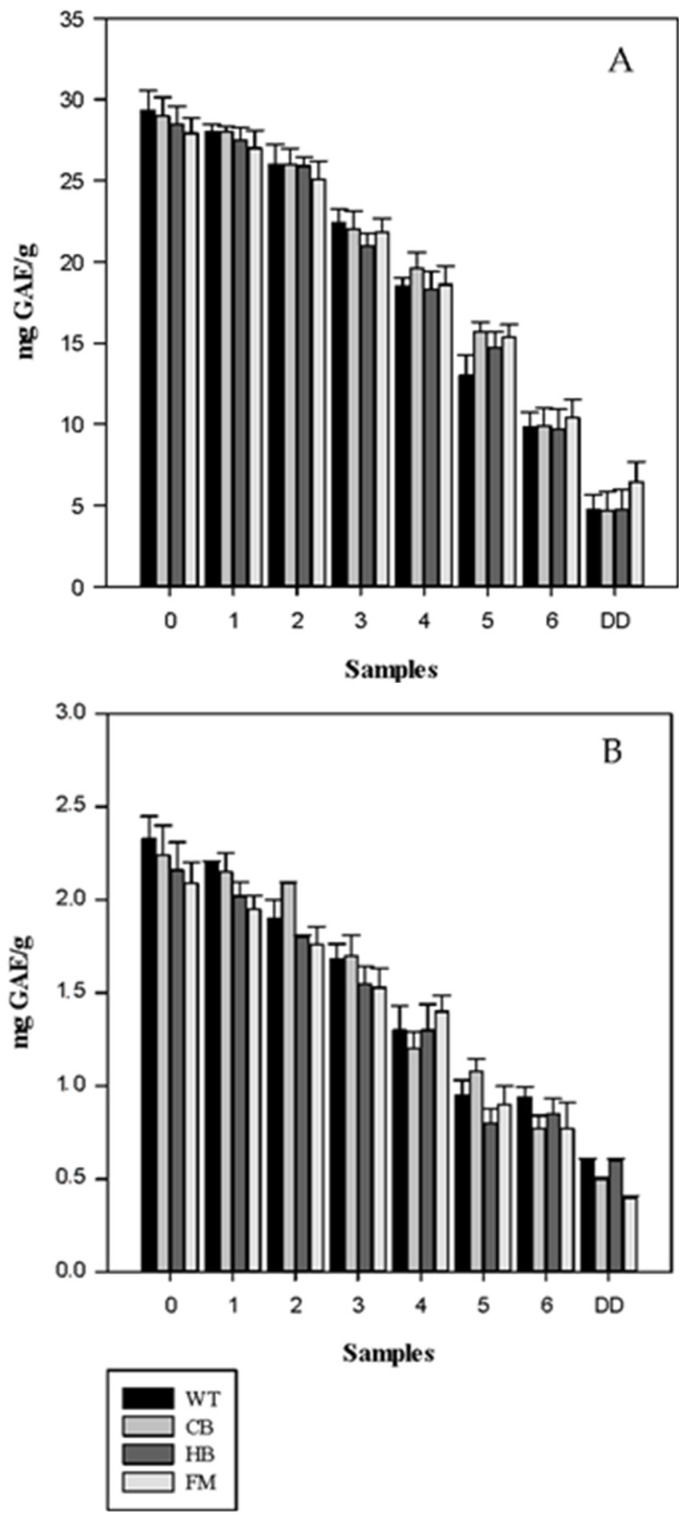
Total phenolic content in natural (NS, panel (**A**)) and blanched (BS, panel (**B**)) almond skin before and after simulated human digestion. Values are expresses in mg GAE/g and they represent mean ± SD of three different experiments. 0 to 6: gastric samples (see Table 1 for sampling time). DD: sample post in vitro gastric + duodenal digestion. Water (WT), home-made biscuits (HB), crisp-bread (CB) and full-fat milk (FM).

**Figure 5:**
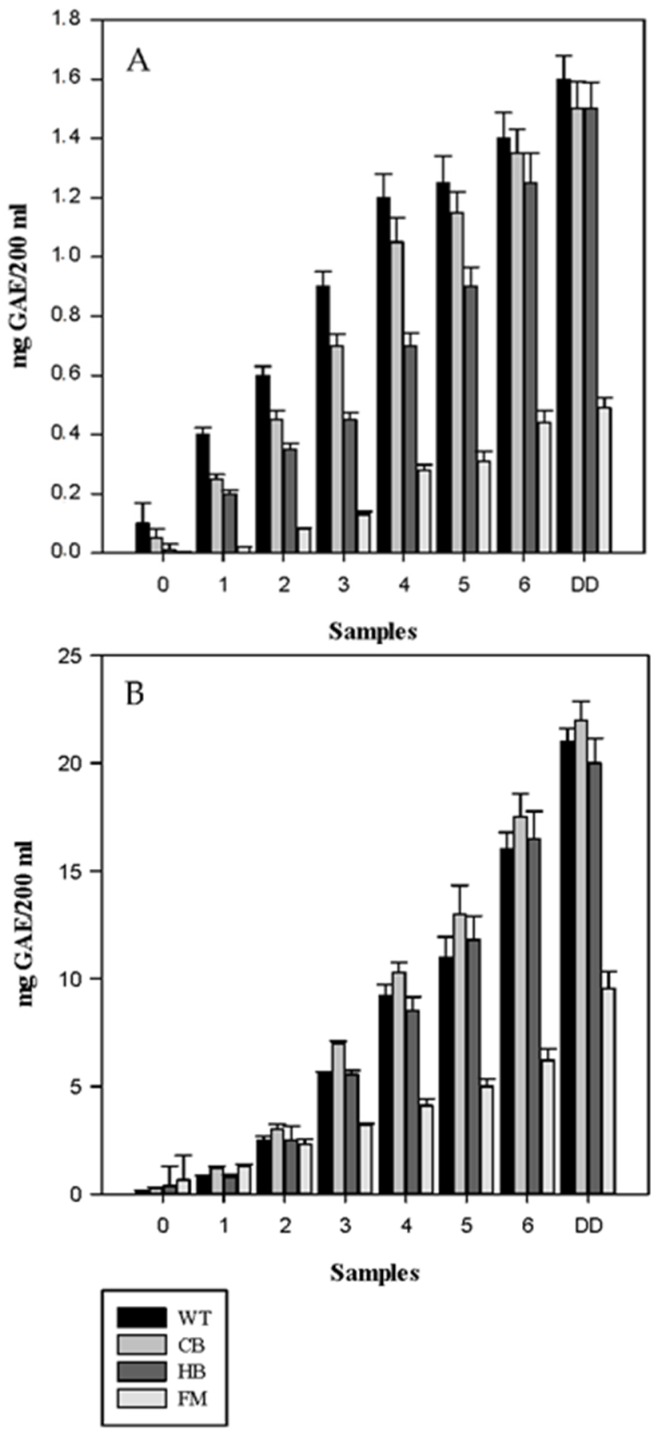
Free total phenols measured in the digestion medium of natural almond skin (**A**) and blanched almond skin (**B**) after in vitro gastric and gastric + duodenal digestion. Values are expressed as mg GAE/200 mL medium. 0 to 6: gastric samples (see Table 1 for sampling time). DD: sample post in vitro gastric + duodenal digestion. Water (WT), home-made biscuits (HB), crisp-bread (CB) and full-fat milk (FM).

The total phenolic content, measured by the Folin-Ciocalteu method, from all the tested meals during digestion is reported in Figure 4A,B. A decrease in the total phenols was observed post in vitro gastric and gastric plus duodenal digestion for both NS and BS in WT, HB, CB and FM. In agreement with the polyphenols release data, significant differences were observed across the four food matrices. A corresponding increase in free total phenols in the digestion medium, expressed as mg GAE/200 mL medium, was observed for WT, HB and CB (Figure 5). However, much lower than expected values of free total phenols were detected in FM, in agreement with the polyphenols recovery data. This data demonstrated that phenolic compounds could bind protein present in the food matrix, thus hindering the antioxidant potential in vitro [21].

**Figure 6:**
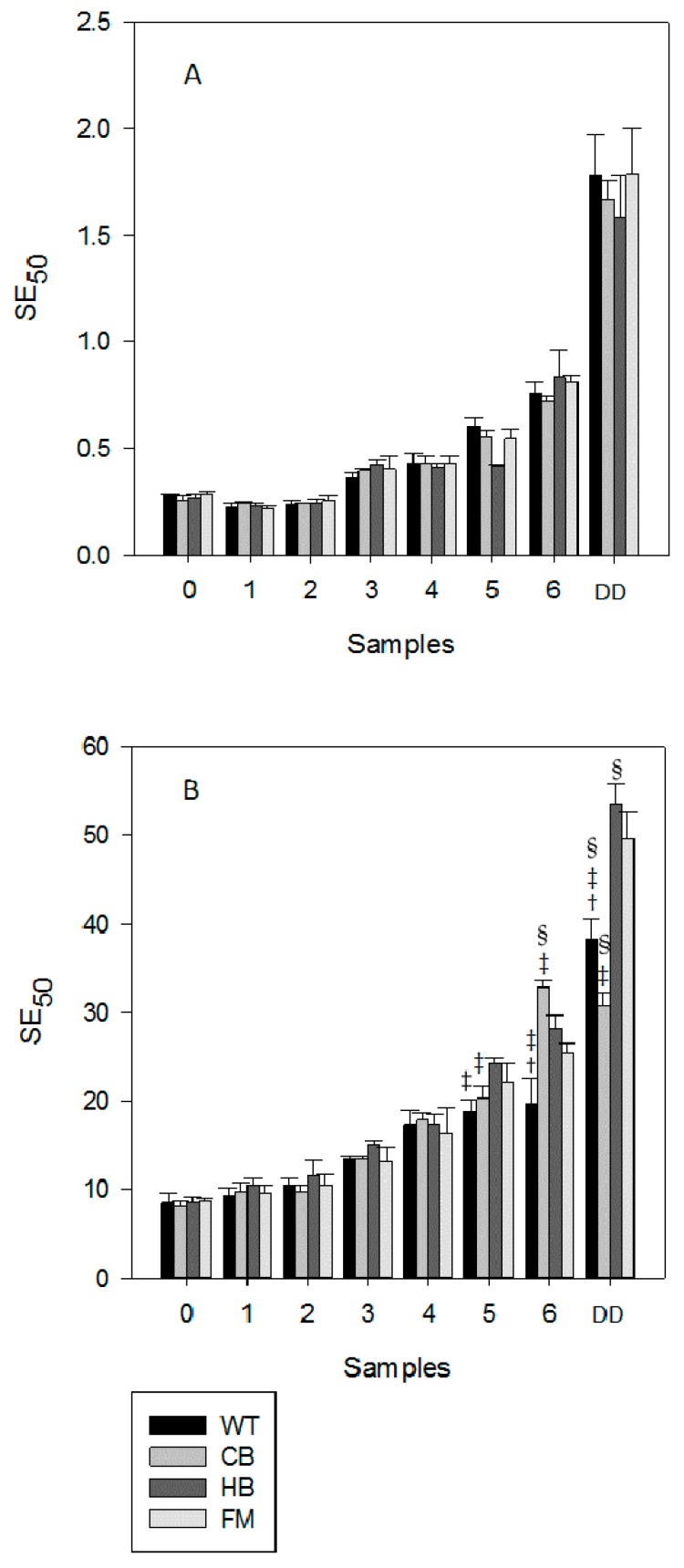
Radical scavenging activity measured in natural (NS, panel (**A**)) and blanched (BS, panel (**B**)) almond skin before and after simulated human digestion. Values are expresses as mg of sample containing the amount needed to scavenge 50 µmol of the initial DPPH solution (SE50) and they represent mean ± SD of three different experiments. 0 to 6: gastric samples (see Table 1 for sampling time). DD: sample post in vitro gastric + duodenal digestion. Water (WT), home-made biscuits (HB), crisp-bread (CB) and full-fat milk (FM). ^†^ *p* < 0.01 vs. CB at the same sampling time; ^‡^ *p* < 0.01 vs. HB at the same sampling time; ^§^ *p* < 0.01 vs. FM at the same sampling time.

**Figure 7:**
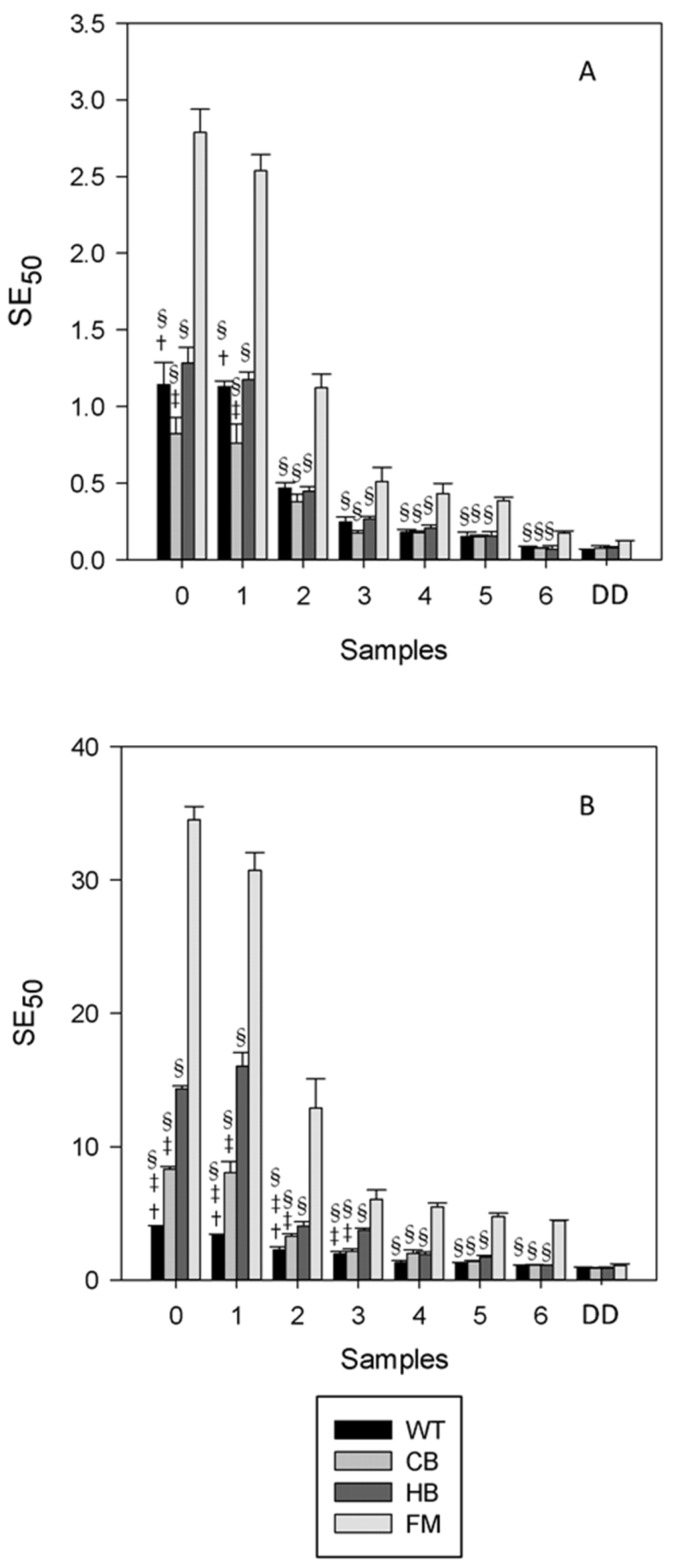
Radical scavenging activity measured in the natural almond skin digestion medium (panel (**A**)) and the blanched almond skin (BS) digestion medium (panel (**B**)) after in vitro gastric and gastric + duodenal digestion. Values are expressed as mg of extract needed to scavenge 50 µmol of the initial DPPH^•^ concentration (SE50). 0 to 6: gastric samples (see Table 1 for sampling time). DD: sample post in vitro gastric + duodenal digestion. Water (WT), home-made biscuits (HB), crisp-bread (CB) and full-fat milk (FM). ^†^ *p* < 0.01 vs. CB at the same sampling time; ^‡^ *p* < 0.01 vs. HB at the same sampling time; ^§^ *p* < 0.01 vs. FM at the same sampling time.

In agreement with the total phenolic content, the radical scavenging activity, measured by DPPH, was lower after digestion (Figure 6). As shown in panel A, no statistically significant difference was observed in NS across all matrices; however in panel B, a statistically significant difference in WT vs. CB, HB and FM for sample 6 (last gastric sample) was observed. A corresponding increase of the antioxidant status was detected in the digestion medium, with the exception of FM; a statistically significant difference (*p* < 0.01) was observed in BS digestion media when comparing FM vs. WT, CB and HB and in NS when comparing FM vs. WT and CB (Figure 7).

## 4. Discussion

The data presented here has demonstrated that bioaccessibility of polyphenols from almond skin was significantly affected by the type of food matrix used. Given the lack of understanding of the fate of antioxidant compounds in the human body, research focused on the bioaccessibility of polyphenols from solid matrices are extremely important in order to better understand the beneficial effect on the host.

We have previously shown that polyphenols from almond skin were bioaccessible in the upper GIT during simulated human digestion in a static system and most of the release occurred in the gastric phase of digestion [1]. However, in the present study, a considerable further loss of polyphenols was observed during the duodenal phase over that detected in the gastric environment across all the food matrices used (Figure 1). The amount of individual polyphenols released from NS and BS are quite different, although a similar rate was detected across the two skin samples. The use of a dynamic model of digestion (DGM), where the digestion products are removed during the time course of the experiment, has likely affected the rate and release of almond skin polyphenols. A similar trend was observed with lipid bioaccessibility from natural raw and roasted almonds after mastication [22]. A study on polyphenols bioaccessibility from apples indicated the release was mainly achieved during the gastric phase (65% of phenolics and flavonoids), with a slight increase (<10%) during intestinal digestion [23].

In vitro digestion of the cocoa insoluble water fraction, source of polyphenols, lead to a 51% release of the total phenols from the insoluble material, without a reduction of the total antioxidant capacity [24].

In our previous study investigating bioaccessibility of bioactives from pistachios, more of 90% of polyphenols were released in the gastric compartment, with little or no increase in the duodenal phase [12]. The lower % of bioaccessibility in almonds (Figure 1) could be due to the properties of their cell walls, which is known to affect lipid and protein bioaccessibility in the gut [22,25]. Almond skins contain high amount of dietary fiber and several cell wall bound phenolics, including *p*-hydroxybenzoic acid, vanillic acid and *t*-ferulic acid [17]. We have previously shown that complex carbohydrates present in dietary fiber can directly interact with antioxidants and therefore interfere with their bioaccessibility in the gut [26,27]. Furthermore, the polyphenols structure plays a crucial role in relation to their adsorption, which was improved by low degree of hydroxylation and reduced by methylation or methoxylation. An increased degree of polymerization determined enhanced absorption for certain polyphenolic classes, including procyanidins [28]. However, the role of glycosylation still remains controversial [13,28]. Dietary fiber can reduce fat bioaccessibility in the GIT and pectin was found to strongly lower β-carotene bioavailability [29]. 

The high dietary fiber content in almond skin, as well as the significant amounts of lipids, could have affected the release of phytochemicals, especially in the absence of a food matrix. Dietary fiber could also reduce the rate of antioxidant absorption by physically trapping the bioactive compounds within its matrix in the chyme, thus restricting enzyme diffusion [30]. Therefore, it is hypothesised that certain polyphenols, mainly phenolic acids which are bound to dietary fiber, are not released in the upper GIT but reach the large bowel where they can be metabolised by the gut microbiota. This could also be due to a dietary fiber specific effect on gastrointestinal physiology (e.g., motility and/or secretion) [28]. The polyphenols-carbohydrates interaction could exert positive effects on lipid metabolism and increase the antioxidant activity in the large intestine [13,28]. 

A number of studies have reported on the effects of a food matrix in a simulated gastrointestinal environment: the findings demonstrated that green tea polyphenols were protected more by the interaction with dairy products, which could help maintain their antioxidant activity during digestion [31] and cheese was identified as an effective matrix for polyphenols protection during gastrointestinal digestion [32]. Stanisavljevic et al. [11] have investigated the changes in polyphenols content and antioxidant activity of chokeberry juice subjected to in vitro gastric digestion in the presence of a food matrix: the results demonstrated a decrease in the total phenolic content, anthocyanin content and DPPH radical scavenging activity immediately after addition of the food matrix. However, the fat content in cocoa samples increased the released of phenolic compounds during duodenal digestion [33]. Lesser et al. [34] have shown that high fat content in meals could either enhance or reduce the absorption of certain flavonoids; polyphenols could also affect the fat adsorption process at the emulsification stage by a direct interaction with phosphatidylcholine or by incorporation within the lipid layer, thus leading to physicochemical property changes of emulsions directly related to lipase activity and fat adsorption decrease, as suggested for tea polyphenols [28]. Moreover, several studies suggested that polyphenols were able to create a positive antioxidant environment at the gastrointestinal level fighting the harmful products of lipid peroxidation [28]. All these interactions could contribute to the well-known beneficial effects of polyphenols.

Another important aspect to discuss is the polyphenols ability to bind proteins, thus affecting the amino acids availability and leading in some cases to protein denaturation (e.g., α-amylase, trypsin, lysozyme) or to their lower digestibility (e.g., β-lactoglobulin) [28,35]. This often affects enzyme activity in a positive way (α-amylase inhibition which could be connected to the prevention of dental injuries) or negative way (when digestive enzymes are involved). It is known, in fact, that protein-polyphenol interactions might influence their adsorption, even though proteins could be carriers of polyphenols through the gastrointestinal tract, thus protecting them from oxidative reactions and increasing their availability at the large intestine [28,35].

In the present study, the fat and protein content in the milk matrix have significantly lowered the release of phenolic acids and flavan-3-ols after simulated human digestion from both NS and BS compared with water, whereas the home-made biscuits decreased bioaccessibility of flavonols (Figure 3). In a recent study [36] the addition of milk decreased the total phenolic, flavonoid and anthocyanin content, although it had no effect on the polyphenols being absorbed in vitro. 

It has also been suggested, with respect to what is mentioned above, that the presence of digestible carbohydrates, lipids and other antioxidant compounds may have a beneficial impact on polyphenols bioaccessibility [37]. This could explain the high bioaccessibility detected when almond skins were incorporated in bread. 

Even if the bioaccessibility of each antioxidant differs greatly, the potential synergistic effect amongst polyphenols could affect their bioactivity and influence on glycoprotein transporters across the mucosal epithelium. It is well established that a variety of factors, including chemical structure, food matrix, digestion enzymes and interaction with the gut microbiota can directly influence polyphenols bioaccessibility and rate of absorption [38]. A number of studies have indicated some unique technological strategies, including micro-encapsulation, which increase polyphenols bioavailability and therefore increase their beneficial health benefits [39,40,41]. 

## 5. Conclusions

In summary, the results of the present study indicate that the presence of a food matrix had a significant effect on polyphenol bioaccessibility from almond skin in both the gastric and duodenal environments. The dietary fiber present in almond skin may act as barrier to prevent a total release of phytochemicals during digestion. Further studies are warranted in order to investigate absorption of bioactives, which could explain the beneficial health effects associated with almond consumption.

## Figures and Tables

**Table 1:** Simulated human digestion parameters.

Matrix	DGM 1	DGM 2	DGM 3	DGM 4	DGM 5	DGM 6	DD	TDT
	Sampling Time (min)							
Water	4	8	12	16	20	24	120	144
Home-made biscuit	4	8	12	16	20	24	120	144
Crisp bread	5	10	15	20	25	30	120	150
Full-fat milk	6	12	18	24	30	36	120	156

DGM = Gastric sample; DD = Duodenal digestion; TDT = Total digestion time.

**Table 2:** Baseline polyphenols content of natural skins and blanched skins in water (W); home-made biscuits (HB); crisp-bread (CB) and full-fat milk (FM). Values were given as µg/g and represent averages (±SD) of triplicate measurements.

**Natural Skin**					
Sample	Phenolic acids	Flavonols	Flavan-3-ols	Flavanones	Total phenols
W	2.15 ± 0.11	14.31 ± 1.05	2.37 ± 0.18	3.43 ± 0.22	22.26
HB	17.85 ± 1.02	115.30 ± 8.32	20.82 ± 1.65	30.44 ± 2.21	184.41
CB	12.53 ± 0.89	83.89 ± 4.22	14.72 ± 1.12	21.78 ± 1.44	132.92
FM	2.07 ± 0.12	13.95 ± 1.22	2.15 ± 0.12	3.22 ± 0.17	21.39
**Blanched Skin**					
Sample	Phenolic acids	Flavonols	Flavan-3-ols	Flavanones	Total phenols
W	0.31 ± 0.02	1.28 ± 0.05	0.62 ± 0.03	0.22 ± 0.02	2.43
HB	2.25 ± 0.14	9.82 ± 0.59	5.18 ± 0.21	1.73 ± 0.102	18.98
CB	1.78 ± 0.12	7.24 ± 0.35	3.65 ± 0.25	1.31 ± 0.09	13.98
FM	0.29 ± 0.01	1.19 ± 0.08	0.60 ± 0.02	0.19 ± 0.01	2. 27

## Abbreviations

The following abbreviations are used in this manuscript:

NS	natural almond skin
BS	blanched almond skin
CB	crisp bread water
WT	water
FM	full-fat milk
HB	home-made biscuits
NSWT G	natural almond skin in water post in vitro gastric digestion
NSHB G	natural almond skin in home-made biscuits post in vitro gastric digestion
NSCB G	natural almond skin in crisp-bread post in vitro gastric digestion
NSFM G	natural almond skin in full-fat milk post in vitro gastric digestion
NSWT G + D	natural almond skin in water post in vitro gastric plus duodenal digestion
NSHB G + D	natural almond skin in home-made biscuits post in vitro gastric plus duodenal digestion
NSCB G + D	natural almond skin in crisp-bread post in vitro gastric plus duodenal digestion
NSFM G + D	natural almond skin in full-fat milk post in vitro gastric plus duodenal digestion
BSWT G	blanched almond skin in water post in vitro gastric digestion
BSHB G	blanched almond skin in home-made biscuits post in vitro gastric digestion
BSCB G	blanched almond skin in crisp-bread post in vitro gastric digestion
BSFM G	blanched almond skin in full-fat milk post in vitro gastric digestion
BSWT G + D	blanched almond skin in water post in vitro gastric plus duodenal digestion
BSHB G + D	blanched almond skin in home-made biscuits post in vitro gastric plus duodenal digestion
BSCB G + D	blanched almond skin in crisp-bread post in vitro gastric plus duodenal digestion
BSFM G + D	blanched almond skin in full-fat milk post in vitro gastric plus duodenal digestion
